# Nanomedicine: Application Areas and Development Prospects

**DOI:** 10.3390/ijms12053303

**Published:** 2011-05-19

**Authors:** Houria Boulaiz, Pablo J. Alvarez, Alberto Ramirez, Juan A. Marchal, Jose Prados, Fernando Rodríguez-Serrano, Macarena Perán, Consolación Melguizo, Antonia Aranega

**Affiliations:** 1 Department of Human Anatomy and Embryology, Institute of Biopathology and Regenerative Medicine (IBIMER), School of Medicine, University of Granada, Granada 18071, Spain; E-Mails: pablo.alvarez.exts@juntadeandalucia.es (P.J.A.); alrari80@gmail.com (A.R.); jmarchal@ugr.es (J.A.M.); jcpradois@ugr.es (J.P.); fernrs@ugr.es (F.R.-S.); melguizo@ugr.es (C.M.); 2 Department of Health Sciences, University of Jaén, Jáen 23071, Spain; E-Mail: mperan@ujaen.es

**Keywords:** nanomedicine, nanostructures, early diagnosis, drug delivery

## Abstract

Nanotechnology, along with related concepts such as nanomaterials, nanostructures and nanoparticles, has become a priority area for scientific research and technological development. Nanotechnology, *i.e*., the creation and utilization of materials and devices at nanometer scale, already has multiple applications in electronics and other fields. However, the greatest expectations are for its application in biotechnology and health, with the direct impact these could have on the quality of health in future societies. The emerging discipline of nanomedicine brings nanotechnology and medicine together in order to develop novel therapies and improve existing treatments. In nanomedicine, atoms and molecules are manipulated to produce nanostructures of the same size as biomolecules for interaction with human cells. This procedure offers a range of new solutions for diagnoses and “smart” treatments by stimulating the body’s own repair mechanisms. It will enhance the early diagnosis and treatment of diseases such as cancer, diabetes, Alzheimer’s, Parkinson’s and cardiovascular diseases. Preventive medicine may then become a reality.

## 1. Introduction

The term nanotechnology refers to the ability to measure, design and manipulate materials at atomic, molecular and supramolecular level in order to understand, create and apply structures and systems with specific functions attributable to their size. Nanotechnology classically refers to matter in the size range of 1–100 nm, but it is often extended to include materials below 1 μm in size. A key goal is to assemble nanoparticles and integrate them into ordered structures in order to obtain useful materials. Although new, the advent of nanomaterials was forecast as long ago as 1959 by Richard P. Feynman [1]. Nanotechnology has been embraced by multiple industrial sectors for application in the field of electronic storage systems [2], biotechnology [3], magnetic separation and preconcentration of target analytes and targeted drug delivery [4,5], and as vehicles for gene and drug delivery [2,4,5]. Advances in nanotechnology have led to the development of new nanomaterials whose physiochemical properties differ from those of their larger counterparts due to their higher surface-to-volume ratio. These novel properties make them excellent candidates for biomedical applications, given the range of biological processes that occur at nanometer scale [6]. Nanotechnology is a new discipline of science and engineering that has led to innovative approaches in many areas of medicine. Its applications in the screening, diagnosis, and treatment of disease are collectively referred to as “nanomedicine”—an emerging field that has the potential to revolutionize individual and population-based health this century [7]. It is now possible to provide therapy at a molecular level with the help of nanoparticles, treating diseases and adding to our understanding of their pathogenesis. Nanomedicine can be considered a refinement of molecular medicine, integrating innovations in genomics and proteomics on the road to a more personalized medicine.

The impact of nanotechnology in medicine can mainly be seen in diagnostic methods, drug-release techniques and regenerative medicine.

Diagnostic methods are essential for the early detection of diseases to enable their prompt treatment, minimizing possible damage to the rest of the organism. The importance of imaging methods to diagnose, treat and follow up cancer, cardiovascular and neurological patients is well known. Diagnostic techniques based on the use of nanoparticles offer higher sensitivity and assist the early detection of disease, offering a better prognosis and greater possibilities of successful treatment [8]. Once the diagnosis is established, the fight against the disease begins, with medicaments playing a major role. There are numerous obstacles to the development of novel drugs against disease. Conventional drugs suffer from the major limitation of adverse effects, the result of the non-specificity of their action, and from a lack of effectiveness due to improper or ineffective dosages, e.g., in cancer chemotherapy and anti-diabetic therapy. Nanotechnology offers the possibility of designing novel drugs with greater cell specificity and new drug-release systems that act selectively on specific targets and protect the drug from degradation *en route*. This allows the administration of smaller but more effective doses, minimizing adverse effects. Nanotechnology can also be used to optimize drug formulations, increasing drug solubility and altering the pharmacokinetics to sustain the release of the drug, thereby prolonging its bioavailability. The diverse platforms of nanotechnology can be utilized to develop more sophisticated, cell-targeted therapies and to combine different drugs into a single nanotherapeutic agent for synergistic therapeutic benefits [9].

Regenerative medicine has been proposed as a highly promising approach to numerous degenerative diseases. To this end, nanomedicine employs gene therapy, cell therapy, tissue engineering, biomaterials and signaling molecules.

## 2. Nanomedicine Applications

**Figure 1.**
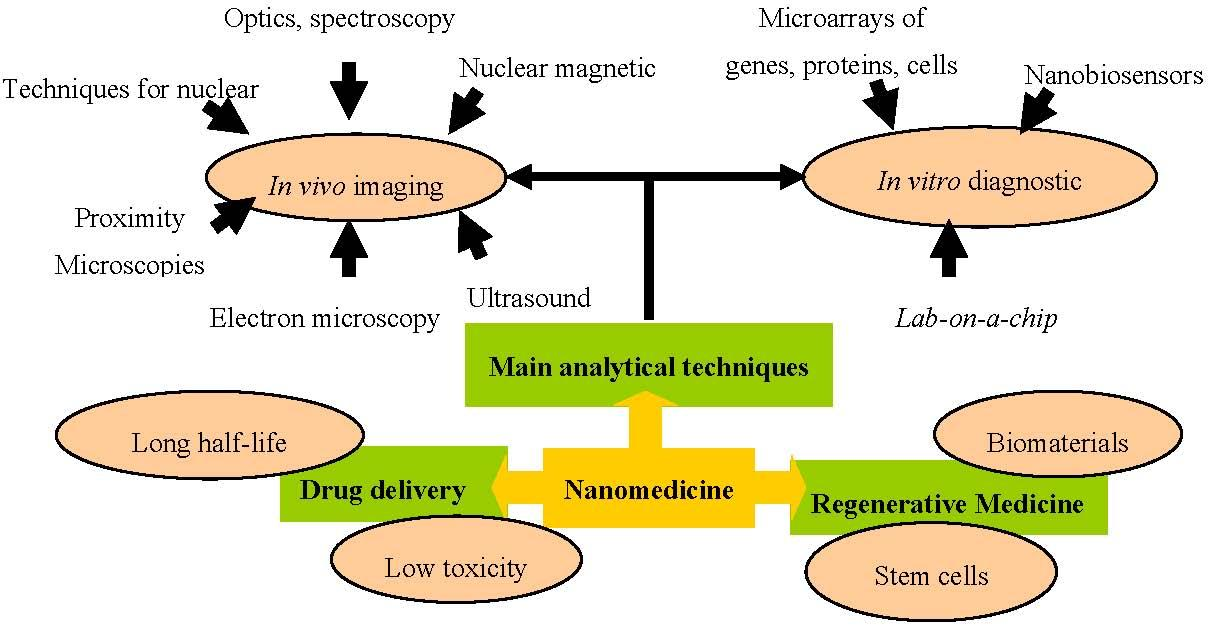
Nanomedicine application areas.

Nanomedicine applications are grouped below in three interrelated areas: analytical/diagnostic tools, drug delivery and regenerative medicine (Figure 1).

### 2.1. Analytical and Diagnostic Tools

The limitations of current diagnostic technology mean that some diseases can only be detected when at a very advanced stage. Nanodiagnostics, defined as the use of nanotechnology for clinical diagnostic purposes [10,11], was developed to meet the demand for increased sensitivity in clinical diagnoses and earlier disease detection.

The application of micro and nanobiotechnology in medical diagnostics can be subdivided into two broad categories: *In vitro* diagnostic devices and *in vivo* imaging. Research in this field is highly multi-disciplinary and there are close relationships among diagnostics, drug release and regenerative medicine, which are described in the following sections.

#### 2.1.1. *In Vitro* Diagnostic Devices

The grounds for modern medicine were already laid in the middle of the 19th century with the recognition of the cell as the source of health and disease. Basic research to yield a better understanding of the highly complex working of cells became mandatory in medicine. The resulting improvements in methods to characterize cells or cell compartments *in vitro* (e.g., optical and luminescence microscopy, scanning probe microscopy, electron microscopy and imaging mass-spectrometry) have been critically important for the development of nanomedicine.

The miniaturization and integration of different functions in a single device, based on nanotechnology-derived techniques, have led to a new generation of devices that are smaller, faster and cheaper, require no special skills and give accurate readings. They require much smaller samples, implying less invasive and traumatic sample extraction methods, and deliver more complete and more accurate biological data from a single measurement. The use of these devices in research has become routine and has improved our understanding of the molecular basis of disease and helped to identify new therapeutic targets. *In vitro* diagnostic devices include nanobiosensors, microarrays, biochips of different elements (DNA, proteins or cells) and lab-on-a-chip devices.

##### 2.1.1.1. Nanobiosensor

A nanobiosensor is defined as a compact analysis device that incorporates biological (nucleic acid, enzyme, antibody, receptor, tissue, cell) or biomimetic (macrophage-inflammatory proteins, aptamers, peptide nucleic acids) recognition elements [11,12]. Interaction between the compound or microorganism of interest and the recognition element produces a variation in one or more physical-chemical properties (e.g., pH, electron transfer, heat, potential, mass, optical properties, *etc*.) that are detected by the transducer. The resulting electronic signal indicates the presence of the analyte of interest and its concentration in the sample. These sensors can be electronically gated to respond to the binding of a single molecule. Prototype sensors have been successfully used to detect nucleic acids, proteins and ions. They can operate in liquid or gas phase, opening up an enormous variety of downstream applications. These detection systems use inexpensive low-voltage measurement methods and detect binding events directly, so there is no need for costly, complicated and time-consuming chemical labeling, e.g., with fluorescent dyes, or for bulky and expensive optical detection systems. As a result, these sensors are inexpensive to manufacture and portable [10]. Hence, nanobiosensors are revolutionizing the *in vitro* diagnosis of diseases and have major implications for human health. They allow healthcare professionals to simultaneously measure multiple clinical parameters using a simple, effective and accurate test. These devices are also ideal for high-throughput screening and for the detection of a single disease in various samples or of various diseases in a single sample [13].

##### 2.1.1.2. Microarrays

The microarray is another diagnostic device that is becoming a standard technology in research laboratories worldwide. Since their first application, microarray technologies have proven productively functional in almost all areas of biomedical research [14–21]. The emergence of this new tool has allowed investigators to address previously intractable problems and identify novel potential therapeutic targets. They are using microarray technology to identify cardinal aspects of growth and development and explore the underlying genetic causes of numerous human diseases [14]. Microarray-based studies have enormous potential in the exploration of diseases such as cancer [15] and in the design and development of new drugs [16]. Microarrays have been widely applied in the study of various pathological conditions, including inflammation [17], atherosclerosis [18], breast cancer [19,20], colon cancer [21] and pulmonary fibrosis [22]. As a result, functions have been assigned to previously unannotated genes, and genes have been grouped into functional pathways [23,24].

Several types of microarray have been developed for different target materials, which can be DNA, cDNA, mRNA, protein, small molecules, tissues or any other material that can be quantitatively analyzed. DNA microarray technology has progressed rapidly over the past 10 years and allows the large-scale quantification of gene expression. A DNA array consists of a large number of DNA molecules orderly arranged on a solid substrate to form a matrix of sequences in two dimensions [25]. cDNA microarrays and oligonucleotide microarrays, called ‘expression chips’, are used for microarray expression analysis, *i.e.*, to determine the level or volume of expression of a given gene. Single nucleotide polymorphism (SNP) microarrays detect mutations or polymorphisms in a gene sequence [26]. This technology is used to test an individual for disease expression patterns and to determine whether or not individuals are susceptible to a disease. Microarray comparative genomic hybridisation [27] is employed to seek genomic gains and losses or changes in the number of copies of a gene implicated in a disease. Protein microarrays are comprised of protein detectors (usually antibodies) arranged systematically over a glass slide and allow the investigation of expression profiles and the precise definition of protein functions in relation to biological processes [28]. Tissue microarrays represent one of the most valuable approaches in the microarray field, because they allow the simultaneous analysis of protein, RNA and DNA expression in multiple tissue samples [29].

The main applications of microarrays in human health are listed below [30].

- *Gene expression analysis*, used to determine gene expression patterns and simultaneously quantify the expression of a large number of genes, permitting comparison of their activation between healthy and diseased tissues.
- *Detection of mutations and polymorphisms,* allowing the study of all possible polymorphisms and the detection of mutations in complex genes.
- *Sequentiation*, used to sequence short DNA fragments (sequencing of long DNA fragments has not yet proven possible, although they can be used as quality controls).
- *Therapy follow-up*, allowing evaluation of genetic features that may affect the response to a given therapy.
- *Preventive medicine,* developing knowledge on the genetic features of diseases in order to treat and prevent them before symptom onset.
- *Drug screening and toxicology,* analyzing changes in gene expression during the administration of a drug, as well as localizing new possible therapeutic targets and testing for associated toxicological effects.
- *Clinical diagnosis*, allowing the rapid identification of pathogens by employing the appropriate genetic markers.

In conclusion, molecular diagnosis is a fast-growing field. Analysis of global expression by microarray techniques simultaneously reveals the state of thousands of genes of diseased cells. These approaches offer a more accurate diagnosis and risk assessment for various diseases, leading to a more precise prognosis and new therapeutic approaches. The ultimate reach of microarray technology will be achieved with its entry into the physician’s clinic as a routine diagnostic tool [31].

##### 2.1.1.3. Lab-on-a-Chip

The latest *in vitro* diagnostic development derives from the integration of several functions in a single device. Lab-on-a-chip integrates one or several laboratory functions on a single chip ranging from only a few millimeters to a square centimeter in size [32] and incorporates sample preparation, purification, storage, mixing, detection and other functions. Its development was based on advances in microsystem technologies and in the field of microfluidics on the design of devices that use microscopic volumes of sample. The chips use a combination of phenomena, including pressure, electroosmosis, electrophoresis and other mechanisms to move samples and reagents through microscopic channels and capillaries, some as small as a few dozen nanometers.

Lab-on-a-chip has many applications in medicine and biology. These devices are likely to have a significant socio-economic impact, bringing sophisticated analytical tools to Third World countries, rural areas and resource-poor regions. Advantages of their use include the extremely rapid analysis of samples containing fluid volumes that can be less than a picoliter, the high degree of automation, cost savings due to the low consumption of reagents and samples and their portable and disposable nature. Lab-on-a-chip is used in real-time polymerase chain reaction [33] and immunoassays [34] to detect bacteria, viruses and cancers. It can also be used in blood sample preparation to crack cells and extract their DNA [32]. Lab-on-a-chip may soon play an important role in efforts to improve global health, especially with the development of point-of-care testing devices. Infectious diseases that are treatable in developed nations are often deadly in countries with limited healthcare resources. In some cases, healthcare clinics possess a drug to treat a given disease but lack the diagnostic tool to identify the patients who should receive it. Many researchers believe that lab-on-a-chip technology will be the key to powerful new diagnostic instruments. The goal is to create microfluidic chips that will allow healthcare providers in poorly-equipped clinics to perform diagnostic tests (e.g., immunoassays and nucleic acid assays) with no laboratory support. One active research line on the lab-on-a-chip addresses the diagnosis and management of HIV infections [35]. Around 40 million people are infected with HIV in the world, yet only 1.3 million receive antiretroviral treatment and around 90% of HIV-infected individuals have never been tested for the disease. This is largely because its diagnosis requires measurement of the number of CD4+ T lymphocytes in the blood by means of flow cytometry, a complicated technique that requires trained technicians and expensive equipment that are not available in most developing regions.

#### 2.1.2. *In Vivo* Imaging

Imaging systems can be grouped according to the energy used to derive the visual information (X-rays, positrons, photons or sound waves), the spatial resolution (macroscopic, mesoscopic or microscopic) or the type of information obtained (anatomical, physiological, cellular or molecular). Unlike classic imaging diagnosis with computed tomography (CT), magnetic resonance imaging (MRI) or ultrasounds, nano-imaging or molecular imaging includes techniques designed to obtain molecular data to identify the causes of the disease *in vivo* rather than its eventual consequences. Nanotechnology has produced advances in imaging diagnosis, developing novel methods and increasing the resolution and sensitivity of existing techniques. Although these systems have emerging recently and only some of them are in clinical and preclinical use, they have made it possible to study human biochemical processes in different organs *in vivo*, opening up new horizons in instrumental diagnostic medicine. These systems include positron-emission tomography (PET), single-photon-emission CT (SPECT), fluorescence reflectance imaging, fluorescence-mediated tomography (FMT), fiber-optic microscopy, optical frequency-domain imaging, bioluminescence imaging, laser-scanning confocal microscopy and multiphoton microscopy [36].

Imaging diagnosis has gained importance over the years and is now an indispensable diagnostic tool for numerous diseases, including cancer, cardiovascular diseases and neurological syndromes. The main benefits of molecular imaging for *in vivo* diagnosis lie in the early detection of disease and the monitoring of disease stages, e.g., in cancer metastasis [37], supporting the development of individualized medicine and the real-time assessment of therapeutic and surgical efficacy. An ideal imaging modality should be non-invasive, sensitive, and provide objective information on cell survival, function and localization. MRI, CT, PET) and SPECT are the most widely used and studied modalities in cancer patients [36,38,39] Overall, nuclear imaging by PET or SPECT offers greater sensitivity (>5 × 10^3^ cells) [40] but is limited by the lack of anatomical context [41], whereas MRI provides accurate anatomical detail but no data on cell viability and shows poor sensitivity (>10^5^ cells) [42]. Although none of these modalities is ideal, MRI is the preferred option for cellular tracking [39,43,44] By detecting proton relaxations in the presence of a magnetic field (1.5 Tesla [T]-3 T for clinical imaging), it yields tomographic images with excellent soft tissue contrast and can locate the cells of interest in the context of the surrounding milieu (oedema or inflammation) [45,46] without the use of harmful ionizing radiations (the case with CT, PET or SPECT). In addition, MRI offers a longer tracking window in comparison to PET and SPECT, which are limited by the decay of the short-lived radioactive isotopes.

In parallel to the development of imaging techniques, intense research has been fuelled by the need for practical, robust and highly sensitive and selective detection agents that can address the deficiencies of conventional technologies. New contrast agents, used to increase the sensitivity and contrast of imaging techniques are increasingly complex and formed by synthetic and biological nanoparticles. Nanoparticles possess certain size-dependent properties, particularly with respect to optical and magnetic parameters, which can be manipulated to achieve a detectable signal [47]. The primary event in most nanoparticle-based assays is the binding of a nanoparticle label or probe to the target biomolecule that will produce a measurable signal characteristic of the target biomolecule. A probe that is to function in a biological system must be water-soluble and stable and have minimal interaction with the surrounding environment. For fluorescence readouts, the probe should ideally have a high fluorescence quantum yield and minimal photobleaching rates in order to generate a detectable signal [48]. The most promising nanotechnologies for clinical diagnosis include quantum dots (QDs), gold nanoparticles and cantilevers, whose main features are summarized in Table 1.

### 2.2. Drug Delivery

One of the most important nanotechnology applications developed over the past decade have been nanovehicles, nanoscale compounds used as a therapeutic tool and designed to specifically accumulate in the sites of the body where they are needed in order to improve pharmacotherapeutic outcomes. The main objective of this application is to increase therapeutic effectiveness while obtaining lower toxicity rates. Hence, nanodrugs and nanodiagnostics have been developed to increase bioavailability profiles, enabling the administration of lower doses and thereby minimizing the adverse reactions found with conventional drugs in clinical practices and increasing the quality of patient health [49]. Table 2 compiles some drugs using nanocarriers and their route of administration.

In the field of cancer therapy there are a lot of clinical applications based on nanotechnologies, with a major development in drug delivery systems. The reason for the rise in nanotechnology applications in medicine is the prospect of improving effectiveness by the biological targeting of drugs in current clinical use [50]. In cancer treatments, nanoparticles are usually administered by intravenous injection, travelling in the blood stream and passing through biological barriers (cell membranes) of the organism in order to reach and activate their molecular targets [51]. One of the main objectives of nanotechnology is overcome the shortcomings of classical chemotherapy, including the multiple drug resistance mechanisms that make this treatment ineffective in a high percentage of cancer cases. The other problem of conventional anticancer therapies is the non-specific action of the drugs, leading them to damage both tumor and non-tumor cells in a state of division.

Nanoparticles can overcome the side effects of conventional therapies by the following means: (1) sustaining drug release over time; (2) so-called “passive enhanced permeability”, targeting the effect to tumor tissue; (3) targeting the cell surface with the use of ligands related to endosomal uptake and membrane disruption; (4) permitting release of the drug in the cell cytoplasm; and (5) protecting the drug from enzymatic degradation [52,53]. The main goals of drug delivery design are: (i) to decrease the side effects of conventional therapy by decreasing drug concentration in normal tissues; (ii) to enhance the pharmacokinetics and pharmacodynamics profiles; (iii) to allow intravenous drug administration by increasing drug solubility; (iv) to minimise drug loss in transit and maximize drug concentration in the tumor; (v) to improve drug stability by avoiding drug degradation; (vi) to achieve optimal cellular uptake and intracellular delivery; and (vii) to ensure biocompatibility and biodegradability [54].

Alternative methods have been developed to increase the effectiveness of drug delivery using the passive or active targeting capacities of nanovehicles. The passive targeting form of drug delivery consists of transporting nanoparticles through leaky tumor capillary fenestrations within the tumor mass to reach cells by convection or passive diffusion. Convection is related to the movement of molecules through organic fluids and is the predominant transport mode for a high percentage of molecules across large pores. However, compounds with a low molecular weight (e.g., oxygen) are most frequently transported by diffusion, in which molecules cross the cell membrane along a concentration gradient, with no energy cost to the cell. In fact, convection through the tumor mass is poor, due to interstitial hypertension; therefore, diffusion is the main type of drug transport used, accumulating nanoparticles with drug by the so-called Enhanced Permeability and Retention (EPR) effect. Nanoparticles are able to achieve an optimal EPR effect if they can evade the immune response, and they remain highly stable in the blood stream until reaching the tumor. The EPR effect is a physiological process based on the capacity of long-circulating nanoparticles to penetrate through the leaky tumor-formed vasculature and degrade within the tumor, releasing the drug and achieving a local concentration in solid tumors that is several-fold higher than that obtained with free drugs [55].

Very high local concentrations of drug-loaded nanoparticles have been obtained within tumors, from 10- to 50-fold higher in comparison to normal tissues in a time period of 1–2 days. The achievement of these drug concentrations in the tumor requires nanoparticles to possess the following characteristics: (a) nanoparticle size between 10 and 100 nm; (b) a neutral or anionic nanoparticle surface charge to prevent elimination by the kidneys; and (c) the ability to avoid opsonization and phagocytosis, which destroy foreign material *via* the reticuloendothelial system [54,56].

Active targeting using nanoparticles as the delivery system allows a specific area of the body to be targeted, avoiding one of the drawbacks of current chemotherapy, *i.e.*, toxic effects in non-malignant organs [57]. Studies are being carried out on the attachment of targeting ligands on the nanoparticle surface, enabling specific binding of the nanoparticle to receptors on the tumor cell surface. Ligands that bind to specific overexpressed receptors are selected. The types of ligand used include antibodies, antibody fragments or non-antibody (peptidic) ligands. Over the past few years, considerable interest has developed in the numerous antigens present on tumor cells. For instance, HER2 receptor has been proposed as a therapeutic target. HER2 gene, also known as c-erbB2 and neu, encodes a 185-kDa transmembrane glycoprotein receptor that belongs to the ErbB family of growth factor receptors with intrinsic tyrosine kinase activity, whose members exist in homodimer and heterodimer form when activated [58]. Trastuzumab is a humanized antibody designed to specifically recognize the HER-2 receptor and was approved by the FDA in 1998 for the treatment of metastatic breast cancer. Different studies have shown that normal cells can express moderate amounts of HER2 target antigen, while it is overexpressed in tumors in certain patients, and this difference between healthy tissues and tumors allows the effective use of this antibody in patients [59].

**Figure 2.**
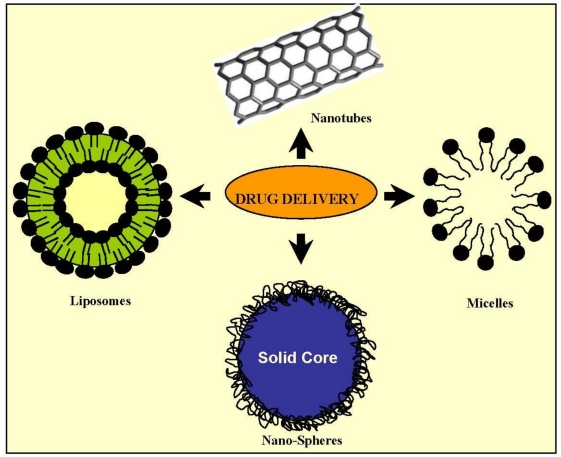
Some drug delivery systems.

Fibroblast growth factor receptors (FGFRs) have also become potential targets for drug delivery and cancer therapy. Fibroblast growth factors (FGFs) are small peptide growth factors that play an important role in tumor growth and angiogenesis because of their high affinity to heparin. FGFRs are upregulated *versus* normal tissues in numerous tumor cells and tumor neovasculature *in situ* [60]. One candidate as a targeting ligand for tumor cells is the peptide KRTGQYKLC (bFGFp), which could interact with FGFR1 by binding to bFGF. The epidermal growth factor receptor (EGFR) is a receptor tyrosine kinase over-expressed on many human cancer cells surface, making it a target for anticancer drug delivery. Liposomes targeting this receptor promoted the effective intracellular delivery of doxorubicin to tumor cells, yielding superior anti-tumor effects in different *in vivo* assays. In previous studies, the peptide GE11 was identified as a novel ligand with high affinity towards EGFR and proved effective to mediate targeted liposome delivery to EGFR-positive tumors *in vivo* [61]. Investigations are ongoing into drug delivery systems based on nanoparticles with different geometry, compositions and surface modifications (Figure 2). They are providing researchers with an enormous collection of nanoparticles with promising applications [62].

#### 2.2.1. Micelles

A micelle is an aggregate of amphiphilic surfactant molecules spontaneously created on immersion in water, usually forming spherical vesicles. They contain a hydrophobic core in which hydrophobic substances, such as pharmacological compounds, can be introduced for release in different parts of the organism. A micelle typically comprises a hydrophilic or polar charged “head” group and a hydrophobic “tail”, normally composed of a hydrocarbonated portion of long fatty acids. The characteristics of the surfactants that make up the micelle determine its size [63].

#### 2.2.2. Nanoemulsions

Nanosized emulsions are composed of a mixture of two-phase insoluble liquids, in which vesicles in the dispersed phase are surrounded by the continuous phase. Different types of surfactants are used to stabilize the emulsion, preventing the dispersed phase from coalescing into a macroscopic phase [64]. Many emulsions used in drug delivery systems can be formulated on a nanosize scale. In these cases, the aqueous phase conform the continuous phase, and the drug is often carried in (or is itself) the non-aqueous liquid phase of the emulsion. Generally, the surfactant molecules used to stabilize micelles are the same as those forming the structural part of the nanoemulsions [65].

#### 2.2.3. Solid Nanoparticles

This structure presents different characteristic features such as: (a) the material present in the centre of the particle forms a solid core at least at room temperature; (b) Solid nanoparticles not necessarily present in a spherical form. The geometry may change, presenting angular forms. This characteristic is particularly visible if the nanoparticle consists of crystals of a protein or another therapeutic agent. To avoid flocculation a surfactant is usually required to stabilize the nanoparticle [65].

#### 2.2.4. Dendrimers

These are usually regularly-branched three-dimensional structures with a treelike form and a molecule as the central core. Branching units emerge from the central molecule by polymerization or are synthesized from the periphery and terminate at the central molecule. Branch lengths have steric limitations and dendrimer forms are sphere-shaped, with low molecular size but high molecular weight. These structures are used to transport drugs in two ways: (a) by attaching drug molecules to functional groups on the dendrimer surface; or (b) by enclosing the drugs in the dendritic channels within the sphere. The well-defined structure, size monodispersity, surface functionalization capability and stability of these nanoparticles make them a promising vehicle system for various agents (e.g., genes and anticancer drugs) by complexation or encapsulation [66,67]. One example is the formation of dendrimers with polymers such as polyamidoamine (PAMAM), which confer stability, availability and tolerability. The drug is contained in a central cavity and can be entrapped in channels between dendrons [23].

#### 2.2.5. Liposomes

The effectiveness of liposomes as drug vehicles is related to their pharmokinetics and depends on the physicochemical conditions, e.g., size, surface charge, membrane lipid packing, steric stabilization, dose, and administration route [68]. A major advantage of liposomes is their long persistence in the blood, favoring the delivery of their contents to target tissues. Liposomes with diameters of 100–300 μm accumulate around tumors and are not subject to rapid clearance by the reticuloendothelial system [69]. Liposomes are vesicular nanostructures formed by a lipid bilayer composed of phospholipid and cholesterol molecules (structural components of cell membranes) characterized by extended, two-dimensional and clearly separated hydrophilic and hydrophobic regions. The hydrophilic portions of bilayer lipids are directed towards aqueous phases (external and internal), whereas the hydrophobic portions of both lipid layers are directed towards one another, forming an internal hydrophilic compartment that can encapsulate water-soluble drugs [70]. Actually there is a type of liposome well studied in clinical, the pegylated liposomes, which forms a water shell on the liposomal surface and provides a steric barrier to the liposomes for avoiding interactions with plasma proteins, resulting in escape from trapping by the reticuloendothelial system [71]. One example is liposomal-encapsulated doxorubicin, which has an elimination half-life of 55 h in comparison to only 0.2 h for doxorubicin in free form. The drugs are solubilized and protected from enzymatic degradation and inactivation either by physical entrapment within the nanoparticle or by conjugation with constituent components. Encapsulation into the liposomal carrier also causes a significant reduction in the most significant adverse side effect of doxorubicin, such as cardiotoxicity [72].

### 2.3. Regenerative Medicine

Tissue engineering brings together principles and innovations from engineering and the life sciences for the improvement, repair or replacement of tissue/organ function. Since its inception, this multidisciplinary field has been governed by the generic concept of combining cell, scaffold (artificial extracellular matrix) and bioreactor technologies in the design and fabrication of neo-tissues/organs [73]. Every tissue or organ in our body is composed of parenchymal cells (functional cells) and mesenchymal cells (support cells) contained within an extracellular matrix to form a microenvironment, and these microenvironments collectively form our tissues and organs. In terms of the development and maintenance of tissues and organs, our body is the “bioreactor”, exposing the microenvironment of the cell and extracellular matrix to biomechanical forces and biochemical signals. The ultimate goal is to enable the body (cellular components) to heal itself by introducing a tissue engineered scaffold that the body recognizes as “self” and uses to regenerate “neo-native” functional tissues [74]. Furthermore, the demand for organs for transplantation far exceeds the supply, and the construction of organs by regenerative therapy has been presented as a promising option to address this deficit. Nanotechnology has the potential to provide instruments that can accelerate progress in the engineering of organs [75]. Achievement of the more ambitious goals of regenerative medicine requires control over the underlying nanostructures of the cell and extracellular matrix. Cells, typically microns in diameter, are composed of numerous nanosized components that all work together to create a highly organized, self-regulating machine. Cell-based therapies, especially those based on stem cells, have generated considerable excitement in the media and scientific communities and are among the most promising and active areas of research in regenerative medicine [76]. The pace of research could be accelerated by the creation of multi-functional tools to improve the monitoring and modification of cell behavior. While nanomedicine is primarily focused on cancer-related research, the application of nanotechnology has considerable potential in cell-based therapies for regenerative medicine, e.g., in localizing, recruiting and labeling stem cells to begin the regeneration process [75].

## 3. Conclusions

Nanotechnology is an emerging interdisciplinary field that combines biology, chemistry and engineering. It is expected to lead to major advances towards individualized medicine, improving the sensitivity and specificity of existing techniques to discover and detect biomarkers and developing novel nanodiagnostic instruments. This would allow earlier and more personalized diagnosis and therapy, improving the effectiveness of drug treatments and reducing side effects. In addition, nanoparticles are a promising platform technology for the synthesis of molecular-specific contrast agents.

## Figures and Tables

**Table 1.** Comparison of quantum dots (QDs), cantilevers and gold nanoparticles.

**Feature**	**QDs**	**Cantilevers**	**Gold nanoparticles**
**Structure**	Semiconductor nanocrystals typically composed of a semiconductor core encapsulated by another semiconductor shell with a larger spectral band-gap; a third silica shell can be added for water solubility	Nano-machined silicon or a piezoelectric material such as quartz similar to those used in atomic force microscopy	Gold particles in the nanometre size domain; gold nanoshells consist of concentric sphere nanoparticles with a dielectric core (typically gold sulfide or silica) surrounded by a thin gold shell
**Size**	2–10 nm	Nanoscale	2–150 nm (changes in optical properties as a function of size)
**Diagnostic applications**	- *In vitro* diagnosis: immunohistochemistry, infectious agent detection, fluoroimmunoassays, immunoassays, intracellular imaging and tissue imaging. - *In vivo* imaging	DNA and protein (various biomarkers) detection and quantification.	Detection of DNA and proteins (including antibodies)
**Method for detecting**	Fluorometry and several types of microscopy, such as fluorescence, confocal, total internal reflection, wide-field epifluorescence, atomic force, and multiphoton microscopy	Operate either statically, by measuring absolute cantilever deflection, or dynamically, by measuring resonance frequency shifts	Surface plasmon resonance microscopy. Gold particles coated ^with^ silver have strong light-scattering properties and can easily be detected by standard dark-field microscopy with white light illumination
**Advantage**	- Their optical tunability, resistance to photobleaching, excitation of various QDs by a single wavelength of light (for multiplexing), narrow emission band, and exceptional stability of optical properties after conjugation to a biomolecule. - They do not need lasers for excitation. - The instrumentation needed for detection is simple.	- Their sensitivity, compatibility with silicon technology, and capacity for microfluidic integration. - Good potential for high throughput protein screening	Their optical properties, useful for imaging and photothermal therapy. Their surfaces, functionalized using various well-characterized chemical moieties (thiols, disulfides, amines)
**Toxicity**	Risk of leakage of toxic core semiconductor materials into host system or into the environment on disposal	No particular toxicity concerns	No particular toxicity concerns

**Table 2.** Some drugs using nanocarriers and their administration routes.

**Compounds**	**Nanocarrier**	**Application**
CPX-1 irinotecan	Liposome	Systemic
DNA (gene therapy)	Solid lipid nanoparticles	Systemic
Cancer vaccine	Immunostimulatory acid-degradable microparticles	Subcutaneously
Camptothecin	Polymeric nanoparticles	Systemic
Tamoxifen citrate	Solid lipid nanoparticles	Systemic
Pilocarpine hydrochloride	Polymeric nanoparticles	Systemic
Clotrimazole	Solid lipid nanoparticles and nanostructured lipid carriers	Topical
Clozapine	Solid lipid nanoparticles	Systemic
Coenzyme Q 10	Solid lipid nanoparticles	Topical
Titanium dioxide	Solid lipid nanoparticles	Topical
5-Fluorouracil	Nanostructured lipid carriers	Systemic
Ibuprofen	Solid lipid nanoparticles	Topical
Insulin	Solid lipid nanoparticles	Systemic
Isotretinoin	Solid lipid nanoparticles	Systemic
Ketoconazole	Solid lipid nanoparticles	Topical
Mifepristone	Solid lipid nanoparticles	Systemic
*N*,*N*-Diethyltoluamide (DEET)	Solid lipid nanoparticles	Topical
*N*-dodecyl-ferulate	Solid lipid nanoparticles	Systemic
Oxybenzone	Solid lipid nanoparticles	Topical
Clobetasol propionate	Nanostructured lipid carriers	Systemic
Retinoids	Solid lipid nanoparticles	Topical
Triptolide	Solid lipid nanoparticles	Systemic
Vitamin A	Solid lipid nanoparticles	Topical
MCC465 doxorubicin	mAb-liposome	Systemic
NC-6004 cisplatin	Micelle	Systemic
NK105 paclitaxel	Micelle	Systemic
NK911 doxorubicin	Micelle	Systemic
PK1 doxorubicin	HPMA copolymer	
SP1049C doxorubicin	Micelle	Systemic
Etoposide	Nanostructured lipid carriers	Systemic
Docetaxel	Nanostructured lipid carriers	Systemic
Paclitaxel	Nanostructured lipid carriers	Orally
Paclitaxel	Polymeric nanoparticles	Subcutaneously
